# Thermodynamic Study of *N*-Methylformamide and *N*,*N*-Dimethyl-Formamide

**DOI:** 10.3390/molecules29051110

**Published:** 2024-03-01

**Authors:** Květoslav Růžička, Vojtěch Štejfa, Ctirad Červinka, Michal Fulem, Jiří Šturala

**Affiliations:** 1 Department of Physical Chemistry, University of Chemistry and Technology, Prague, Technická 5, CZ-166 28 Prague, Czech Republic; stejfav@vscht.cz (V.Š.); cervinkc@vscht.cz (C.Č.); fulemm@vscht.cz (M.F.); 2 Department of Inorganic Chemistry, University of Chemistry and Technology, Prague, Technická 5, CZ-166 28 Prague, Czech Republic; sturalaj@vscht.cz

**Keywords:** *N*-methylformamide, *N*,*N*-dimethylformamide, liquid phase, vapor pressure, vaporization enthalpy, heat capacity

## Abstract

An extensive thermodynamic study of *N*-methylformamide (CAS RN: 123-39-7) and *N*,*N*-dimethylformamide (CAS RN: 68-12-2), is presented in this work. The liquid heat capacities of *N*-methylformamide were measured by Tian–Calvet calorimetry in the temperature interval (250–300) K. The vapor pressures for *N*-methylformamide and *N*,*N*-dimethylformamide were measured using static method in the temperature range 238 K to 308 K. The ideal-gas thermodynamic properties were calculated using a combination of the density functional theory (DFT) and statistical thermodynamics. A consistent thermodynamic description was developed using the method of simultaneous correlation, where the experimental and selected literature data for vapor pressures, vaporization enthalpies, and liquid phase heat capacities and the calculated ideal-gas heat capacities were treated together to ensure overall thermodynamic consistency of the results. The resulting vapor pressure equation is valid from the triple point to the normal boiling point temperature.

## 1. Introduction

This work represents a continuation of our efforts to provide reliable thermodynamic data for biogenic compounds, including α,ω-diamines [1], acetamides [2], proteinogenic α-amino acids [3,4], and their N-acetyl amides [5]. It reports new thermodynamic data for *N*-methylformamide (NMF) and *N*,*N*-dimethylformamide (DMF).

NMF and DMF are (given their low molar mass) high boiling compounds, having normal boiling temperatures (*T*_nbp_) of approximately 472 K and 426 K, respectively. The disparity in *T*_nbp_ is attributed to hydrogen bonds in NMF (H-bonds are reported only in mixtures containing DMF, not in pure DMF [6], which is aprotic).The difference in normal temperatures of fusion is even higher (270.6 K for NMF [7] and 212.86 K for DMF [8]). 

NMF contains (–CO) and (–NH) groups, interconnected in the same manner as a peptide bond, playing a central role in biochemistry. The aqueous solution of DMF serves as a model solvent for the interior of proteins [9].

Amides, due to their similarity to proteins, generally serve as model substances for basic research in the investigation of protein conformation, hydration, and stability. Both NMF and DMF are commonly used solvents in peptide synthesis, and they play similar roles in facilitating the chemical reactions involved in building peptide bonds.

Beyond biological applications, NMF and DMF are used on an industrial scale as reactants [10,11] and as excellent solvents for both organic compounds and inorganic electrolytes [12], because of their chemical bifunctionality and high polarity. They find extensive use in the manufacture of, e.g., pharmaceuticals, pesticides, fibers, adhesives, and coatings. Their unlimited miscibility in water and many organic solvents (including alcohols, esters, ethers, ketones, and aromatic hydrocarbons) contrasts with their immiscibility with aliphatic hydrocarbons, which is utilized for separating aromatics from aliphatic components in petroleum refinement [12,13]. 

The prevailing use of DMF is attributed to its superior thermal stability; NMF undergoes chemical transformations at higher temperatures, especially when in contact with residual water [13]. Solutions of salts in DMF serve as fillers for electrolytic capacitors and batteries owing to the high dielectric constant of DMF [12].

As NMF and DMF serve as model substances for studying interactions in biological systems, they have been extensively studied both experimentally [6,14,15,16,17] and computationally [18,19,20,21]. Both compounds could be used in parametrizing molecular dynamics force fields (FFs) for an accurate representation of peptide bond behavior, or for FF verification. The ability of an FF to correctly capture the energetics and dynamics of peptide bonds is essential for simulating larger and more complex peptide structures.

In addition to quantities describing properties of a single molecule (e.g., bond lengths and orientation, spatial arrangement, charge distribution), macroscopic physico-chemical quantities, such as excess properties of mixtures containing NMF or DMF, and, in particular, densities and vaporization enthalpies of pure compounds, are necessary for the parameterization of FFs (and/or for their validation). While liquid densities can be readily obtained for NMF and DMF using, for example, vibrating tube densitometers, the accurate determination of vaporization enthalpies for high boiling substances is challenging, especially near the room temperature, i.e., in the low-pressure region. At the same time, enthalpies of vaporization (when combined with enthalpies of solution at infinite dilution) can yield enthalpies of solvation, especially hydration. These quantities are decisive for understanding the nature of solute–solvent interaction, both in water and organic solvents [22].

Therefore, both NMF and DMF deserve new vapor pressure measurements near room temperature (enabling the calculation of vaporization enthalpies) and a more complex processing of related thermodynamic properties, leading to consistent data with low uncertainties. Densities and vapor pressures are essential also for parametrization of equations of state (e.g., SAFT family EOS), which are aimed for industrial use.

Calorimetry, static manometry, and theoretical calculations for properties in the state of ideal gas were used to obtain new data. These were combined with selected literature data that passed thermodynamic consistency tests. As a result, vapor pressures, vaporization enthalpies, and heat capacities of liquid and ideal gas were established, with a focus on biologically relevant ambient temperature region.

## 2. Results and Discussion

### 2.1. Thermogravimetry

While DMF is thermally stable even at elevated temperatures, thermal stability of NMF at higher temperatures (and especially in presence of water traces) represent a well-known issue [13]. Moreover, NMF is rather hygroscopic [23]. This instability might affect the measured quantities published in the literature (for example, scatter of published vapor pressures is unusually high) as well as values in this work. We have, therefore, performed a TGA experiment coupled with mass spectrometry to evaluate NMF stability under dynamic argon atmosphere. We do not want to discuss all possible mechanisms of NMF decomposition; however, there are two pathways which we took into account.

1. Reaction between two molecules of NMF to yield *N*-methyl-diformylamine and gaseous methylamine. This path does not require any further reagent and might occur even in pure NMF.
2. Reaction with trace water (or any nucleophilic impurities, e.g., alcohols), which yields formic acid (or its derivates) and methylamine.

**Figure 1:**
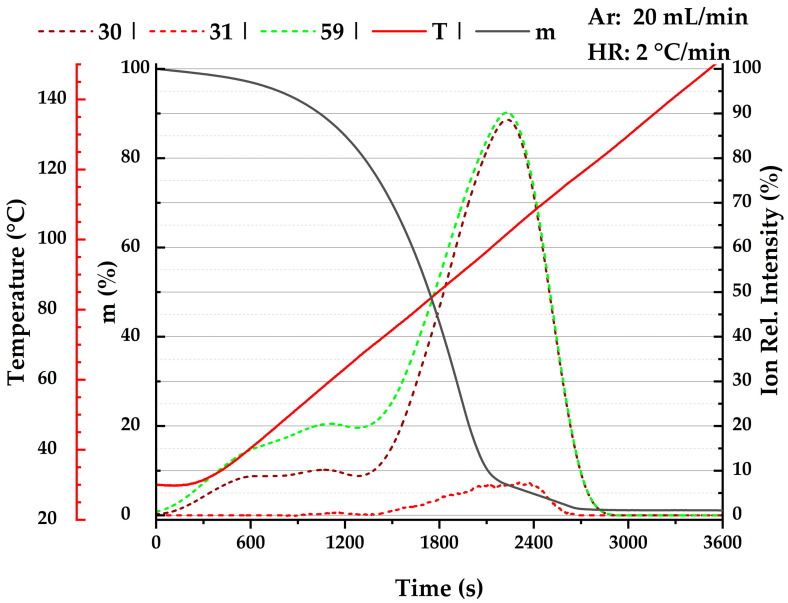
TG-MS spectrum of NMF at heating rate 2 K min^−1^. Black solid line is mass, red solid line is temperature, dashed green line is NMF ion with *m*/*z* 59, dashed red line is methylamine ion with *m*/*z* 31, and dashed brown line is *m*/*z* ion 30.

The mass spectrum of NMF, according to NIST database [24], is composed of the following major ions, which are relevant for our analysis: 59 (100%), 31 (2%), 30 (54%); followed by other ions: 58 (24%), 29 (13%), 28 (34%), and 15 (7%). Ion 59 represents the molecular peak, and 31 represents the methylamine fragment. The methylamine spectrum is composed of major ions 31 (65%) and 30 (100%), followed by other ions 29 (21%), 28 (54%), 27 (8%), and 15 (4%). Based on these data, it is clear that one has to compare the ratio of the relative intensity of ions 59 and 31, because the ion 31 has negligible intensity in the case of NMF but very high intensity in the case of methylamine. We also used peak 30 as a reference peak, which is present in both compounds (note that the intensities of methylamine and NMF are not directly comparable). The spectrum containing both TG data and ion intensities is shown in Figure 1 for heating rate 2 K min^−1^; for heating rate 5 K min^−1^, see Figure S1 in the Supporting Materials (SM). Up to 72 °C (heating rate 2 K min^−1^) or 82 °C (heating rate 5 K min^−1^), the peak 31 has almost zero intensity and the ratio between 59 and 30 is almost 2:1, which reflects the data for NMF. When that temperature is reached, the intensity of peak 31 is gradually increasing and the 2:1 ratio between peaks 59 and 30 is not preserved, which implies its decomposition into gaseous methylamine and other by-products.

### 2.2. Vapor Pressure

Numerous literature sources providing vapor pressure *p* data for both titular amides can be found (see summary presented in Table 1). The majority of these sources encompass a pressure range spanning from several kilopascals to the *T*_nbp_. The reported values exhibit some scatter, particularly in the case of NMF, possibly attributable to the thermal instability of NMF discussed in the preceding section. 

In the case of DMF, the literature results obtained using ebulliometry (at pressures higher than ca. 10 kPa) seem to be in a reasonable agreement. Below 1 kilopascal, which corresponds to temperatures close to ambient, data are limited, and the measurements conducted in this study using STAT 6 apparatus [47] were intended to address this informational gap. Note that in the case of NMF, measurements of this work could be extended to supercooled liquid state below the normal melting temperature of 270.6 K [7] (see Table 2). 

**Figure 2:**
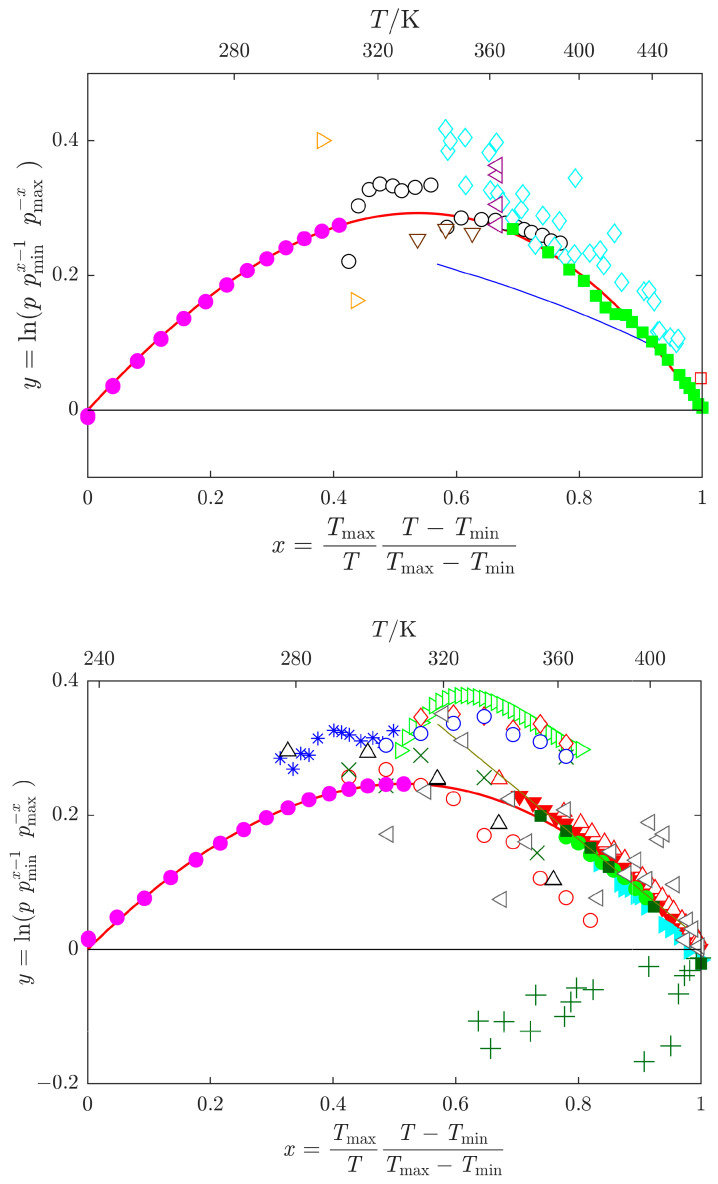
Arc representation [48] of vapor pressure data *p* for *N*-methylformamide (**top**), and *N*,*N*-dimethylformamide (**bottom**). Datasets used in the SimCor method (Section 2.6) are denoted by filled symbols: magenta 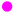, this work; green 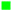, Heinrich et al. [13]; green 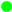, Shealy and Sandler [39]; cyan 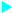, Blanco et al. [43]; dark green 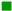, Wang et al. [44]; red 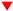, Muñoz et al. [45]. Other datasets: cyan 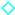, Kortüm and Biedersee [23]; black 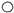, Messow et al. [25]; blue 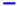, Ushakov et al. [26]; orange 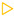, Zielkiewicz [27,28,29]; violet 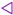, Harris et al. [30]; brown 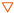, Chen et al. [31]; red 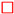, Li et al. [32]; grey 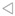, Ivanova and Geller [33]; blue 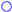, Gopal et al. [34]; black 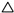, Quitzsch et al. [35]; dark green 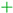, Myasnikova et al. [36] (partially displayed); olive 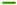, Bludilina et al. [37]; red 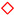, Agarwal and Bapat [38]; olive 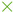, Wilding et al. [40]; red 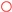, Polishchuk et al. [41]; red 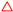, Marzal et al. [42]; green 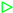, Cui et al. [46]; blue 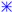, Zaitseva et al. [17]; red 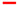, data obtained by SimCor method.

The available data are graphically compared in Figure 2. Since the ln *p −* 1/*T* plot is rather insensitive for the purpose of comparing different vapor pressure datasets, the arc visualization [48] is employed in Figure 2, where the differences are magnified. This approach allows for the identification (and rejection) of obvious outliers prior to any data fitting. Note that for most of the datasets, which differ from the majority of the data in Figure 2, the description of the samples is incomplete (especially in terms of water content).

The selection of which data should be retained or rejected in the final correlation must be made using thermodynamic consistency testing (SimCor method described in Section 3.6). As this testing requires vaporization enthalpies (discussed in Section 2.3) and heat capacities of the liquid and ideal gas (Section 2.4 and Section 2.5), it will be presented later in Section 2.6.

### 2.3. Enthalpies of Vaporization

Calorimetric determination of vaporization enthalpies $\Delta_{l}^{g}H_{m}$ at ambient temperatures presents a challenge, especially for high-boiling compounds. The sole paper reporting calorimetric vaporization enthalpies is by Barone et al. [16], who studied both NMF and DMF. The authors utilized a modified commercial sorption LKB microcalorimeter operating at 298.15 K. A miniaturized custom-made effusion cell was adapted to the microcalorimeter, and vaporization was performed isothermally into a vacuum through a small orifice, allowing a vapor pressure very close to the equilibrium value. The modified calorimeter was tested by measuring the enthalpies of vaporization at 298.15 K of several reference liquid compounds [49] (see Section S2 in the Supplementary Materials for more details). Barone et al.’s work [16] appears to have been carried out meticulously, using a purified and dried sample and a claimed reproducibility of 1 percent. Relative deviation from recommended data [50,51,52] was well below 1 percent (with the exception of rather volatile benzene, see Table S1 in the Supplementary Materials). It is noteworthy, however, that the samples used for calorimeter testing [49] are more volatile than DMF and especially NMF. SimCor method (Section 2.6) will be used to test the consistency of calorimetric $\Delta_{l}^{g}H_{m}$ with vapor pressures and heat capacities. 

In a compilation summarizing phase change enthalpies [53], a paper by Panneerselvam et al. [54] is listed as a source of DMF vaporization enthalpy. However, this work will not be considered for the final regression in our study for two reasons. The first reason is the use of the CGC (Correlation Gas Chromatography) method, an indirect gas-liquid chromatographic method based on an empirical finding that there is a linear relationship between vaporization enthalpies of reference compounds at 298 K and the slope of the logarithm of retention time plotted as a function of 1/T at elevated temperatures (the temperature range of chromatographic measurements in [54] was from 473 K to 513 K). This method was extensively used primarily for the determination of vapor pressures, as it is relatively fast and insensitive to impurities. Our previously published analysis of published CGC results [55] revealed that many approximations and extensive extrapolations used in the CGC method can lead to large systematic errors. The second reason for rejection is that DMF was used by Panneerselvam et al. [54] as a reference compound with a known vaporization enthalpy taken from the paper by Barone et al. [16], and it is therefore not an independent value. The values discussed in this section are summarized in Table 3. 

### 2.4. Liquid Heat Capacities

**Figure 3:**
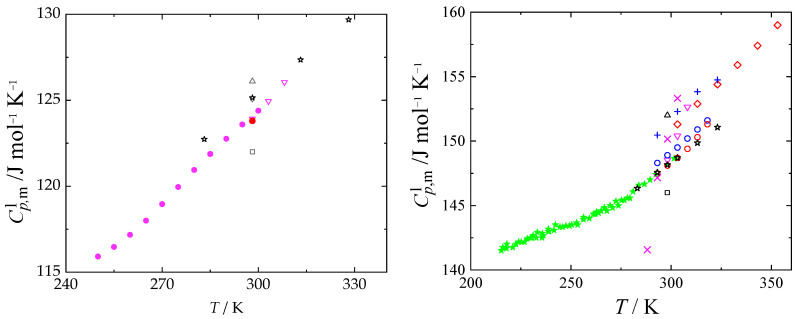
Comparison of available liquid heat capacities $C_{p,m}^{1}$ for *N*-methylformamide (**left**) and *N*,*N*–dimethylformamide (**right**). Data accepted for SimCor method (Section 2.6) are denoted by filled symbols: magenta 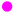, this work; red 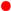, Sköld et al. [14]; green 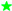, Smirnova et al. [8]. Other da-tasets: black 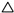, de Visser and Somsen [56]; black 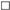, Bonner and Cerutti [57]; black 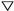, de Visser et al. [58]; black 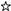, Kolker et al. [59,62]; magenta 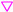, Sharma and Dua [60,61]; blue 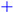, Prasad et al. [63]; magenta 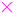, Checoni and Volpe [64]; red 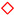, Shokouhi et al. [65]; red 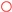, Tyczyńska et al. [66,67]; blue 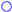, Tyczyńska et al. [68].

Reliable adiabatic liquid heat capacities $C_{p,m}^{l}$ can be found for DMF [8], eliminating the need for any experimental efforts. On the other hand, the literature data for NMF are fragmented, with four sources reporting heat capacity at a single temperature of 298 K, and two sources reporting over a short temperature range (see Table 4). As mutual agreement of literature values was not perfect, we conducted new measurements using Tian–Calvet calorimeter SETARAM Microcalvet. In order to avoid potential issues related to decomposition, as discussed in Section 2.1, we restricted the temperature range to temperatures below 300 K. The continuous cooling method allowed us to measure the heat capacity of supercooled liquid NMF too (down to 250 K). The solidification of the sample made it impossible to evaluate the heat capacities from subsequent measurements in heating mode. As the results for the cooling mode were reproducible (and in accordance with the literature data), they were considered for further treatment; the resulting heat capacities are presented in Table 5 and graphically compared with the literature data in Figure 3.

After the completion of the abovementioned measurements, the experiment was modified to avoid solidification of the sample during cooling. Subsequent heating of the supercooled sample showed a non-monotonic pattern with a local maximum (see Figure S2 in the Supplementary Materials). This interesting (and reproducible) phenomenon would merit further investigation but is beyond the scope of this paper.

**Table 4:** Overview of the literature heat capacities $C_{p,m}^{1}$ of *N*-methylformamide and *N*,*N*-dimethylformamide.

Year	Reference ^a^	*N* ^b^	(*T*_min_ − *T*_max_)/K	$100u_{r}\;(C_{p,m}^{l}$) ^c^	Method
	*N*-methylformamide				
1974	de Visser and Somsen [56]	1	298.15	0.7 ^d^	Isoperibol
1976	Bonner and Cerutti [57]	1	298.15	1.0	Isoperibol
**1976**	**Sköld et al. [14]**	**1**	**298.15**	**0.2 ^d^**	**Drop**
1977	de Visser et al. [58]	1	298.15	1.0	Isoperibol
1992	Kolker et al. [59]	4	283–328	0.05	“Adiabatic” ^e^
2014	Sharma and Dua [60,61]	3	298–308	0.3	Tian–Calvet ^f^
**2024**	**This work**	**11**	**250–300**	**0.6**	**Tian–Calvet**
	*N*,*N*-dimethylformamide ^g^				
1974	de Visser and Somsen [56]	1	298.15	0.3 ^d^	Isoperibol
1976	Bonner and Cerutti [57]	1	298.15	1.0	Isoperibol
1992	Kolker et al. [62]	6	283–323	0.05	“Adiabatic” ^e^
1994	Prasad et al. [63]	4	293–323	nosp	DTA
**2007**	**Smirnova et al. [8]**	**57**	**216–302**	**0.3**	**Adiabatic**
2010	Checoni and Volpe [64]	4	288–303	nosp	solution
2013	Shokouhi et al. [65]	6	303–353	0.2	Hot wire
2014	Sharma and Dua [60,61]	3	298–308	0.3	Tian–Calvet
2019	Tyczyńska et al. [66,67]	6	293–318	0.2	Tian–Calvet
2023	Tyczyńska et al. [68]	6	293–318	0.2	Tian–Calvet

^a^ The data from references written in bold were considered for inclusion in SimCor method (Section 2.6). ^b^ *N* = number of data points. ^c^ *u_r_*($C_{p,m}^{1}$) stands for relative uncertainty of the heat capacity as reported by the authors, unless stated otherwise. ^d^ The absolute uncertainty in heat capacity (in J·K^−1^·mol^−1^) as reported by the authors. ^e^ “Adiabatic shell calorimeter of container type” used primarily for measurement of mixtures. Claimed uncertainty is overoptimistic. There is no ref-erence material for which heat capacities uncertainty is lower than 0.1 percent. ^f^ The Tian–Calvet calorimeter belongs to the class of heat-flux calorimeters; however, its sensitivity is much higher because the sample is surrounded by a large number of thermopiles. For details, see, e.g., Chapter 7.9.2.3 in Sarge et al. [69]. ^g^ Due to sufficient amount of sources reporting liquid heat capacity of *N*,*N*-Dimethylformamide as a function of temperature, some sources containing heat capacity at single temperature are not listed.

**Table 5:** Experimental liquid heat capacities $C_{p,m}^{1}$ of *N*-methylformamide at *p* = (100 ± 5) kPa ^a,b^.

*T*/K	$C_{p,m}^{l}$/J·K^−1^·mol^−1^	*T*/K	$C_{p,m}^{l}$/J·K^−1^·mol^−1^	*T*/K	$C_{p,m}^{l}$/J·K^−1^·mol^−1^
250.0	115.9	270.0	119.0	290.0	122.8
255.0	116.5	275.0	120.0	295.0	123.6
260.0	117.2	280.0	120.9	300.0	124.4
265.0	118.0	285.0	121.9		

^a^ Standard uncertainty *u* is *u*(*T*) = 0.05 K, and the combined expanded uncertainty of the heat ca-pacity is $U_{c}\left(C_{p,m}^{1}\right)=0.006\cdot C_{p,m}^{1}$ (0.95 level of confidence). ^b^ Values are reported with one digit more than is justified by the experimental uncertainty to avoid round-off errors in calculations based on these results.

### 2.5. Ideal-Gas Heat Capacities

The heat capacities of studied compounds in the ideal gaseous state $C_{p,m}^{g0}$ were calculated by a combination of quantum chemical and statistical-thermodynamic calculations, as generally described in Section 3.5. Details specific to the two studied amides are given below.

*N*-Methylformamide. A vibrational assignment is reported by Popov et al. [70] for a single NMF conformer only. Since NMF molecules can form two non-equivalent conformations *cis-* and *trans*-regarded with respect to the mutual position of N-hydrogen and C-hydrogen atoms, we decided to adopt the scaled B3LYP-D3 frequencies for both conformers in all subsequent models to be able to follow the equilibrium conformation mixing model. B3LYP-D3/6-311+G(2df,p) level of theory predicts the *cis* conformer to be more stable which is in agreement with experimental determination [71]. However, the computed enthalpy difference 4.90 kJ∙mol^−1^ at 298 K is significantly underestimated when compared to the experimental value 7.44 kJ∙mol^−1^. The latter value was used in the calculations of conformation mixing models [72]. According to the given enthalpy difference, 95% of molecules are present in the *cis* form at 300 K and 71% at 1000 K, which makes the use of the conformation mixing model appropriate. Reference spectral data on molecular structure can be found in the literature [73]. Principal moments of inertia based on this reference correspond to the less stable *trans* form, calling for an uncertainty revision of the thermodynamic properties based on these values which are listed in a handbook by Frenkel at al. [72]. Thus, we take our newly calculated ideal-gas data for NMF as more reliable. The calculated products of inertia amount to *I*_ABC_^cis^ = 1.004∙10^−135^ kg^3^∙m^6^ and *I*_ABC_^trans^ = 7.405∙10^−136^ kg^3^∙m^6^. The molar mass used to calculate the translation contributions was *M* = 59.0672 g∙mol^−1^. The reduced moments of inertia *I*_r_ and barriers to internal rotation for the methyl rotor amount to *I*_Me_^cis^ = 4.86∙10^−47^ kg∙m^2^, *V*_Me_^cis^ = 0.2 kJ∙mol^−1^ and *I*_Me_^trans^ = 3.95∙10^−47^ kg∙m^2^, *V*_Me_^trans^ = 3.8 kJ∙mol^−1^. The *V*_Me_^trans^ value is in close agreement with a reference value [74] 4.6 kJ∙mol^−1^. Properties of both pure conformers have been computed, and then, the mixing terms were added to evaluate the total thermodynamic properties of NMF, which are listed in Table 6. At 300 K, the contribution of the conformer equilibrium to $C_{p,m}^{g0}$ amounts to 3.0 J K^−1^ mol^−1^, which dominates the difference between our results and the earlier published data [72,74].

*N*,*N*-Dimethylformamide. A complete vibrational assignment by Jao et al. [75] is available for DMF. Since this molecule exists in a single unique conformer, there is no need for following the conformer equilibrium mixing model. We thus employed the experimental frequencies for the RRHA calculations. No experimental data on barriers to internal rotation or moments of inertia have been found for DMF, so calculated values were used for further calculations. Molar mass *M* = 79.0938 g∙mol^−1^ and principal moments of inertia *I*_A_ = 9.447∙10^−46^ kg∙m^2^, *I*_B_ = 2.018∙10^−45^ kg∙m^2^, and *I*_C_ = 2.856∙10^−45^ kg∙m^2^ were used for the calculations. 

The *N*-methyl group adjacent to the carbonyl oxygen atom undergoes an internal rotation with computed barrier *V*_CO-N-Me_ = 4.3 kJ∙mol^−1^ and *I*_CO-N-Me_ = 5.18∙10^−47^ kg∙m^2^, while the other *N*-methyl rotation, neighboring to the carbonyl hydrogen atom, possesses parameters *V*_CH-N-Me_ = 8.7 kJ∙mol^−1^ and *I*_CH-N-Me_ = 5.03∙10^−47^ kg∙m^2^. The calculated thermodynamic properties arising from calculated or experimental vibration frequencies differ by less than 1.5% at ambient temperature, which is a satisfying agreement. All values are listed in Table 6.

### 2.6. Selection of Recommended Data for NMF and DMF Using SimCor Method

The vapor pressures discussed in Section 2.2 are thermodynamically linked, via the Clapeyron equation, to the enthalpies of vaporization (Section 2.3), which, in turn, correlate with the difference between the heat capacities of an ideal gas (Section 2.5) and a liquid (Section 2.4). The SimCor method, explained in detail in Section 3.6, allows for the simultaneous correlation of all these related quantities, utilizing a suitable vapor pressure equation. In this study, we employed the Cox equation, Equation (1).

(i) Vapor pressures. As described in Section 2.2, vapor pressure datasets underwent initial analysis using the arc representation [48]. This analysis revealed outliers, which, being distant from the rest of the data, were excluded from the final correlation (see Figure 2). The remaining vapor pressure datasets were subjected to the SimCor method [76] for consistency, refining the selection of datasets used in the final correlation. For NMF, only two datasets passed thermodynamic tests, with the vapor pressures obtained in this work using the static method proving thermodynamically consistent along with ebulliometric data by Heinrich et al. [13]. Other literature vapor pressure data were either thermodynamically inconsistent with related thermal data [26,31] or exhibited significantly larger scatter than other data available in the same temperature range [23,25].

**Figure 4:**
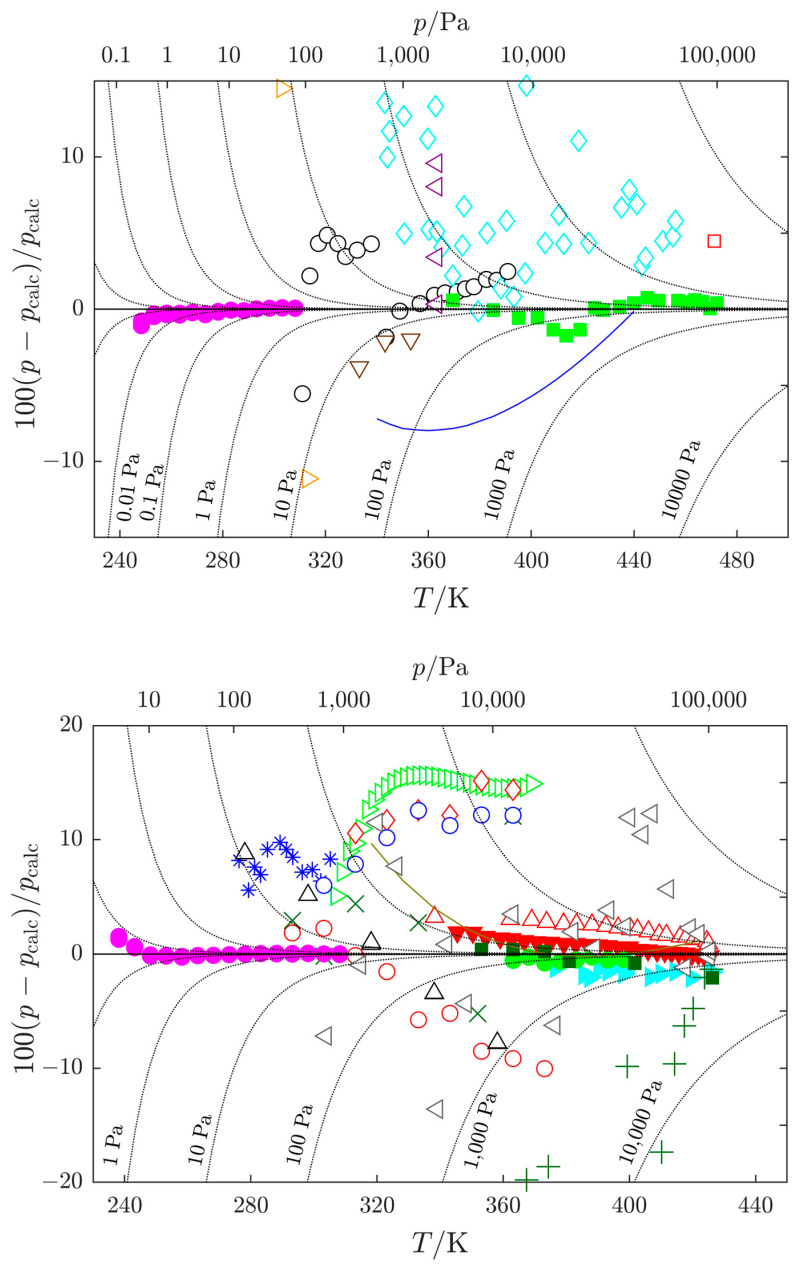
Relative deviations of vapor pressures *p* for *N*-methylformamide (**top**), and *N*,*N*-dimethylformamide (**bottom**) from the recommended values pcalc calculated with the Cox equation, Equation (1), with parameters listed in Table 7. Datasets used in the SimCor method are denoted by filled symbols: magenta 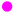, this work; green 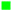, Heinrich et al. [13]; green 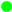, Shealy and Sandler [39]; cyan 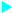, Blanco et al. [43]; dark green 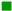, Wang et al. [44]; red 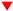, Muñoz et al. [45]. Other datasets: cyan 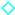, Kortüm and Biedersee [23]; black 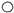, Messow et al. [25]; blue 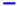, Ushakov et al. [26]; orange 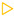, Zielkiewicz [27,28,29]; violet 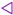, Harris et al. [30]; brown 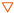, Chen et al. [31]; red 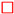, Li et al. [32]; grey 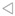, Ivanova and Geller [33]; blue 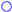, Gopal et al. [34]; black 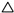, Quitzsch et al. [35]; dark green 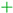, Myasnikova et al. [36] (partially displayed); olive 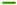, Bludilina et al. [37]; red 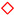, Agarwal and Bapat [38]; olive 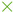, Wilding et al. [40]; red 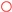, Polishchuk et al. [41]; red 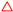, Marzal et al. [42]; green 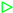, Cui et al. [46]; blue 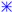, Zaitseva et al. [17]; 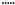, absolute deviations.

**Figure 5:**
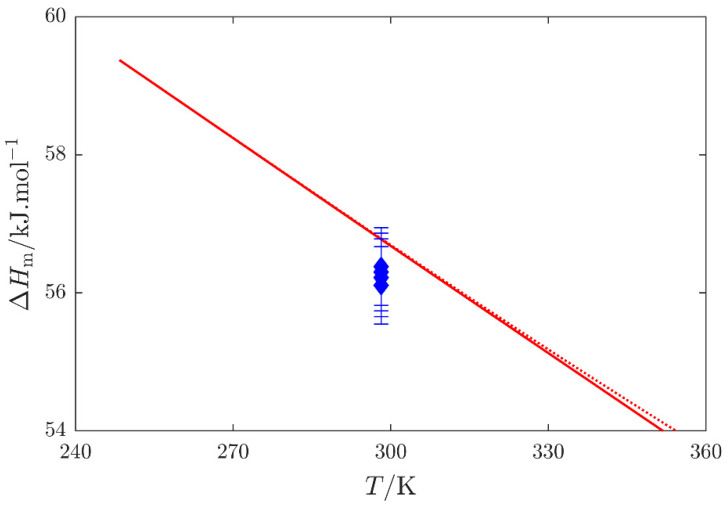

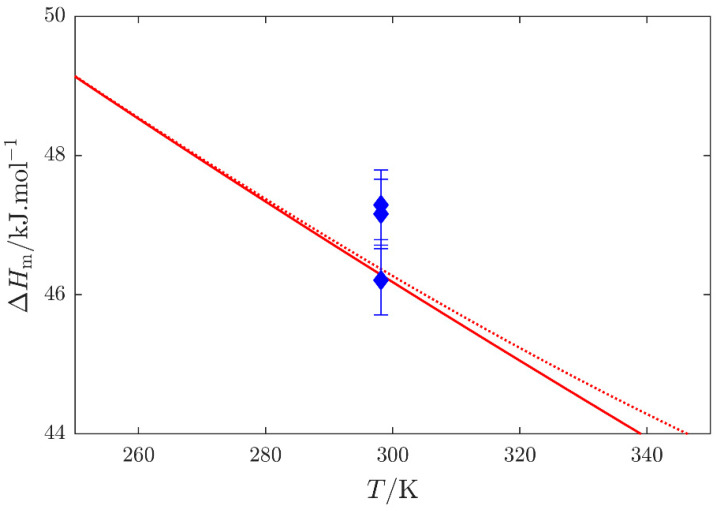
Calorimetric enthalpy of vaporization $\Delta_{1}^{g}H_{m}$: blue 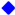, Barone et al. [16]; red 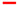, enthalpy of vaporization $\Delta_{1}^{g}H_{m}$, and red 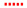 , quantity $\Delta_{1}^{g}H_{m}/\Delta_{1}^{g}Z$ (see Equation (S1) in the Supplementary Materials) obtained by the SimCor method. Datasets published by Barone et al. [16] were used in the SimCor method.

For DMF, due to the abundance of data, more datasets were considered for the final correlation. Besides vapor pressures from this work obtained using the static method, only several datasets obtained using the ebulliometric method at sufficiently high pressures (above approximately 10 kPa) passed thermodynamic testing [39,43,45]. Similar to NMF, other literature vapor pressure data were either thermodynamically inconsistent with related thermal data [42] or showed larger scatter than other data available in the same temperature range [17,38]. It should be noted that the best available vapor pressure data were selected; however, the uncertainty of ebulliometric data exceeds several hundred pascals, an order of magnitude higher than in the case of standard reference compounds, as shown in Figure 4. There might be room for improvement; however, measurements at elevated temperatures are apparently difficult due to the thermal instability of the studied compounds.

(ii) Enthalpy of vaporization (Section 2.3). Calorimetric vaporization enthalpy data published by Barone et al. [16] for both NMF and DMF fitted well within their stated uncertainty with that derived from selected vapor pressures, as shown in Figure 5. 

(iii) Liquid heat capacities (Section 2.4). For NMF, only two datasets [59,61] reported liquid heat capacities as a function of temperature over a relatively narrow temperature range (see Table 4). New measurements in this work extended the available temperature range toward the temperature of melting and even below it. They were in very good agreement with a single value obtained by drop calorimetry [14] (see Figure 3). Datasets [59,61] differed slightly (though still within combined uncertainties) and were not included in the final correlation. In the case of DMF, reliable adiabatic heat capacities were published by Smirnova et al. [8]. Three other datasets [62,66,68], while in reasonable agreement with adiabatic data [8], were not necessary for the final correlation, as heat capacities can be safely included in the final correlation only at temperatures corresponding to vapor pressures lower than approximately 1 kPa (see Section S4 in the Supplementary Materials). 

(iv) Ideal-gas heat capacities (Section 2.5). Due to simplistic approximations used in previously calculated ideal-gas heat capacities for NMF, data of this work were used. This holds true also for DMF, for which there are no literature data for comparison. 

The selected vapor pressure data (given in bold in Table 2) were treated simultaneously with calorimetric enthalpies of vaporization [16], selected liquid heat capacities (given in bold in Table 4), and ideal-gas heat capacities (Table 6), using the SimCor method. The thermal data were employed in the temperature range where the *pVT* correction in Equations (S1) and (S2) in the Supplementary Materials does not significantly impact the SimCor method. For *pVT* corrections, second virial coefficients estimated by the method of Tsonopoulos [77] were used using experimental critical temperatures and pressures [78] and dipole moments [79,80] (see Table S2). 

The Cox equation, Equation (1), was employed in the SimCor method; its parameters are presented in Table 7. Deviations of individual datasets from the SimCor results are shown in Figure 4 and Figure 5 for vapor pressures and vaporization enthalpies, respectively. 

While vapor pressures can be easily calculated using the Cox equation, the calculation of the enthalpies of vaporization using the Clapeyron equation requires evaluation of the appropriate *pVT* correction based on the estimated second virial coefficient *B*. To avoid the need to calculate *B*, the vaporization enthalpies along with the associated uncertainties are listed in Table S3 in the SM for the convenience of the reader.

## 3. Materials and Methods

For calibration purposes and for all the measurements, the international temperature scale ITS-90 was used. Molar masses of the compounds were calculated based on IUPAC recommendations [81]. For the calculations, the molar gas constant *R* = 8.314462618 J K^−1^ mol^−1^ was used [82].

### 3.1. Samples Description

The title amides were of commercial origin. *N*-methyl formamide was distilled at reduced pressure ca. 2.7 kPa under dry atmosphere using spinning band microdistillation column and stored over molecular sieves prior to measurements. DMA was purchased as anhydrous with protecting septum, and due to its high purity (see Table 8), it was used as received.

### 3.2. Thermogravimetry

Thermogravimetric analysis was carried out using a Themys TGA (SETARAM, Caluire, France) linked to a mass spectrometer with electron impact ionization (OMNI Star) at a temperature range between 30 and 200 °C and a heating rate of 2 and 5 K min^−1^. The instrument was purged with argon for one hour before the measurement started, and to equilibrate the temperature at 30 °C. Argon was used as a carrier gas with a flow rate of 100 mL min^−1^ (heating rate 5 K min^−1^) or 20 mL min^−1^ (heating rate 2 K min^−1^). About 20 mg of *N*-methylformamide was used for the analysis. It should be noted that contact of the sample with the laboratory atmosphere was minimized but not completely eliminated.

### 3.3. Vapor Pressures

Vapor pressure measurements were performed using static apparatus with capacitance diaphragm gauges STAT6 [47]. The reader is referred to the original paper [47] for details on its design, calibration, and measurement procedure. The uncertainty for the STAT6 apparatus, i.e., the combined expanded uncertainty (0.95 level of confidence, *k* = 2), of vapor pressure measurement is *U*_c_(*p*/Pa) = 0.005*p*/Pa + 0.05. 

### 3.4. Heat Capacity Measurements

A Tian–Calvet type calorimeter (SETARAM Microcalvet) was used for the measurement of heat capacities in the temperature range from 250 K to 300 K. The heat capacity measurements were carried out by the continuous heating method [83], using the three-step methodology, i.e., the measurement of the sample is followed by the measurement of the reference material (synthetic sapphire, NIST Standard reference material No. 720) and by performing a blank experiment. The saturated molar heat capacities *C*_sat_ obtained in this work are identical to isobaric molar heat capacities $C_{p,m}^{l}$ in the temperature range studied, given the very low vapor pressure of the samples. The combined expanded uncertainty (0.95 level of confidence) of the heat capacity based on measurements of four reference compounds (naphthalene, benzophenone, benzothiazol, and benzoic acid) is estimated to be $U_{c}(C_{p,m}^{l})=0.006C_{p,m}^{l}$.

### 3.5. Theoretical Calculations

Thermodynamic properties of both amides in the ideal gaseous state were calculated using the RRHO model [84] with corrections for internal rotations, the 1DHR model [85,86], and optionally assuming an equilibrium mixture of multiple conformations. Optimization of molecular geometries, fundamental vibration frequencies, and barriers to internal rotations were calculated using the Gaussian 16 software package [87] by the DFT method on the B3LYP-D3/6-311+G(2df,p) level of theory [88,89,90], which has been thoroughly tested in our previous work [91,92]. The calculated fundamental harmonic frequencies were scaled by a double-linear scaling factor (0.9972 − 1.48·10^−5^ ν cm^−1^)/0.960 for frequencies below/above 2000 cm^−1^ [93], developed on experimental vibrational frequencies of *n*-alkanes. We assume the order of the carbon–nitrogen bond in both molecules to be higher than one so that no internal rotation takes place around this bond, being supported by our relaxed potential energy scans predicting the respective barriers to this rotation over 90 kJ∙mol^−1^. 

Reduced moments of inertia of the methyl groups, required in the 1DHR model, have been evaluated according to the formula for symmetric tops by Pitzer [94], based on the B3LYP-D3 optimized molecular geometries. Energy levels of the hindered internal rotations were obtained by solving a one-dimensional Schrödinger equation using our code performing the FGH method [95]. The expected standard uncertainty of calculated $C_{p,m}^{g0}$ does not exceed 2% for low temperatures where $C_{p,m}^{g0}$ were used in the SimCor method (Section 3.6); uncertainty at higher temperatures is likely to be lower. Uncertainty in $S_{m}^{g0}$ amounts to 0.8%. These values are based on statistical evaluation of uncertainties of calculated thermodynamic properties published in our previous papers [92,96].

### 3.6. Simultaneous Treatment of Vapor Pressures and Related Thermal Data (SimCor Method)

The simultaneous correlation of vapor pressures and related thermal properties (SimCor, suggested in a simplified form by King and Al-Najjar [97]) is based on exact thermodynamic relationships, and the procedure must therefore yield reliable results providing that the input data are of reasonable accuracy. A great advantage of this approach is that a single equation can furnish a description of the temperature dependences of several thermodynamic properties, resulting in a set of vapor pressure equation parameters which are valid in a combined temperature range of all input experimental values. The SimCor also provides a test on the consistency of different experimental data (vapor pressures *p*, calorimetrical vaporization enthalpies $\Delta_{l}^{g}H_{m}$, differences in the heat capacities between ideal-gas and liquid phase, $\Delta_{l}^{g}C_{p,m}^{0}=C_{p,m}^{g0}-C_{p,m}^{l},$ where $C_{p,m}^{g0}$ and $C_{p,m}^{l}$ were obtained as described in previous section and from the calorimetric measurements, respectively). The SimCor method has been described in detail in, e.g., [76,98] and was used in our laboratory to develop recommended vapor pressure and thermophysical data for several groups of crystalline and liquid compounds (see, e.g., Mahnel et al. [98] and references therein). Real behavior of the gas phase was approximated through the method of Tsonopoulos [77] using dipole moments and critical temperatures and pressures. The resulting *pVT* corrections are small and well below the normal boiling point, so that even relatively high uncertainty in the estimated *pVT* description has negligible impact on the final results. Note that the method of Tsonopoulos was utilized since it introduces class-specific corrections to the second virial coefficient; however, any amides were not considered during its development. The second virial coefficient was therefore approximated by the equation for ketones, which seem to be most similar molecules, but not forming hydrogen bonds. Thermodynamic equations related to SimCor method are summarized in Section S4 in the Supplementary Materials.

The Cox equation [99] was used within the SimCor procedure to describe the vapor pressures and the linked thermodynamic properties since it requires lower number of adjustable parameters than other equations while keeping a comparable description:

(1) $$\ln\frac{p}{p^{\mathrm{ref}}}=\left(1-\frac{T^{\mathrm{ref}}/K}{T/K}\right)\exp\left(\sum_{i=0}^{2}A_{i}{\left(T/K\right)}^{i}\right),$$

where *p*^ref^ and *T*^ref^ are reference pressure and temperature, respectively, and *A_i_* are the adjustable parameters. 

## 4. Conclusions

In this study, we investigated two biologically and industrially important compounds: *N*-methylformamide and *N*,*N*-dimethylformamide, focusing on biologically important near-ambient temperatures. A literature search revealed that new vapor pressures for both compounds as well as liquid heat capacities for *N*-methylformamide are needed. Additionally, properties in the ideal gaseous state were uncertain or missing. 

To address these gaps, we conducted measurements of vapor pressures and liquid heat capacities using the static method and the Tian–Calvet calorimeter, respectively. Properties of the two compounds in the ideal gaseous state were obtained using quantum chemical calculations and statistical thermodynamics. By simultaneously correlating the aforementioned properties (along with scarce literature vaporization enthalpies and selected literature vapor pressures), we achieved their thermodynamically consistent description along the saturation curve from the melting to the normal boiling temperature. 

It was found that the description could be improved by measurement of vapor pressures above 10 kPa using ebulliometry (not available in our laboratory). Such measurements might, however, be challenging for such high-boiling and thermally not very stable compounds. In the vicinity of the ambient temperature, the uncertainty is low, and the data obtained by the SimCor method can be considered reliable. 

## Figures and Tables

**Table 1:** Overview of vapor pressures *p* of *N*-methylformamide and of *N*,*N*-dimethylformamide.

Year	Reference ^a^	*N* ^b^	(*T*_min_ − *T*_max_)/K	(*p*_min_ − *p*_max_)/Pa	Method
		*N*-methylformamide			
**1961**	**Heinrich et al. [13]**	**19**	**370–472**	**2653–100,405**	**Ebulliometry**
1970	Kortüm and Biedersee [23]	33	343–456	773–101,000	Ebulliometry
1974	Messow et al. [25]	19	311–391	89–6920	Isoteniscope
1996	Ushakov et al. [26]	S ^c^	340–440	532–39,312	Static
1996–1998	Zielkiewicz [27,28,29]	3	303,313	60–95	Static
2003	Harris et al. [30]	4	363	1950–2130	Static
2010	Chen et al. [31]	3	333–353	370–1150	Static/dynamic
2019	Li et al. [32]	1	471	101,325	Ebulliometry
**2024**	**This work**	**39**	**248–308**	**0.3–75**	**Static**
		*N*,*N*-dimethylformamide ^d^			
1961	Ivanova and Geller [33]	22	304–425	666–101,325	Ramsay Young
1968	Gopal et al. [34]	7	303–363	733–14,532	Static
1969	Quitzsch et al. [35]	5	278–358	141–9775	Isoteniscope
1974	Myasnikova et al. [36]	17	331–426	2266–101,325	Ebulliometry
1979	Bludilina et al. [37]	S ^c^	318–423	1784–95,906	Static
1985	Agarwal and Bapat [38]	6	313–353	1366–14,818	Static
**1985**	**Shealy and Sandler [39] ^e^**	**8**	**363–398**	**11,900–44,740**	**Ebulliometry**
1987	Wilding et al. [40]	6	293–363	380–14,520	Static
1988	Polishchuk et al. [41]	9	293–373	376–17,091	Static
1995	Marzal et al. [42]	20	338–425	4600–100,780	Ebulliometry
**1997**	**Blanco et al. [43]**	**13**	**376–426**	**21,400–101,310**	**Ebulliometry**
**2001**	**Wang et al.** [44]	**6**	**353–426**	**8660–101,300**	**Ebulliometry**
**2005**	**Muñoz et al.** [45]	**26**	**346–426**	**6300–101,200**	**Ebulliometry**
2006	Cui et al. [46]	32	307–369	922–18,806	Static
2019	Zaitseva et al. [17]	12	276–305	123–847	Gas saturation
**2024**	**This work**	**45**	**238–308**	**4–930**	**Static**

^a^ Datasets printed in bold were used in the SimCor method (Section 2.6). ^b^ *N* = number of data points. ^c^ S denotes data in the form of equation only. Such datasets are excluded from further fitting as their statistically rigorous treatment is not possible. ^d^ Sources containing one or two vapor pressure points are not listed. ^e^ The value of 12.71 kPa at 368.15 K in Shealy and Sandler [39] is an obvious printing error and a value of 15.71 kPa was used.

**Table 2:** Experimental vapor pressures *p* for *N*-methylformamide and *N*,*N*-dimethylformamide measured with STAT6 apparatus ^a^.

*T*/K	*p* ^b^/Pa	*T*/K	*p* ^b^/Pa	*T*/K	*p* ^b^/Pa
*N*-methylformamide					
248.35	0.32	268.22	2.69	288.19	16.21
248.35	0.32	268.22	2.68	293.18	24.36
248.35	0.32	273.21	4.32	293.18	24.36
253.26	0.57	273.21	4.32	293.18	24.36
253.26	0.57	273.22	4.32	298.17	36.02
253.26	0.57	278.21	6.84	298.17	36.02
258.25	0.97	278.21	6.84	298.17	36.02
258.25	0.97	278.21	6.84	303.15	52.44
258.25	0.97	283.20	10.63	303.15	52.45
263.24	1.63	283.20	10.63	303.15	52.44
263.24	1.63	283.20	10.63	308.14	75.34
263.24	1.63	288.19	16.21	308.14	75.33
268.22	2.68	288.19	16.21	308.15	75.38
*N*,*N*-dimethylformamide					
238.15	3.85	263.15	39.95	288.15	264.71
238.16	3.84	263.15	39.96	288.15	264.77
238.16	3.84	263.15	39.96	288.15	264.85
243.15	6.39	268.15	60.26	293.15	369.48
243.16	6.39	268.15	60.27	293.15	369.48
243.16	6.39	268.15	60.26	293.16	369.49
248.15	10.37	273.15	89.36	298.15	508.91
248.15	10.38	273.15	89.37	298.15	508.93
248.15	10.38	273.15	89.37	298.15	508.74
253.15	16.60	278.15	130.35	303.15	691.81
253.15	16.59	278.15	130.33	303.15	691.79
253.15	16.59	278.15	130.35	303.15	691.83
258.15	25.97	283.15	187.19	308.15	930.12
258.15	25.99	283.15	187.20	308.15	929.99
258.15	25.98	283.15	187.17	308.16	929.99

^a^ The standard uncertainty in the sample temperature measurements is *u*(*T*) = 0.01 K, and combined expanded uncertainty (0.95 level of confidence, *k* = 2) in the vapor pressure measurements is *U*_c_(*p*) = 0.005 *p* + 0.05 Pa. ^b^ Values are reported with one digit more than is justified by the experimental uncertainty to avoid round-off errors in calculations based on these results.

**Table 3:** Summary of vaporization enthalpies $\Delta_{1}^{g}H_{m}$ (in kJ∙mol^−1^) at 298.15 K obtained using calorimetry [16] and indirect chromatographic method [54], which used calorimetric data [16] as input ref-erence values ^a^.

Compound	*N*-Methylformamide	*N*,*N*-Dimethylformamide	*N*,*N*-Dimethylformamide
Literature	Barone et al. [16]	Barone et al. [16]	Panneerselvam et al. [54]
Method	Calorimetry	Calorimetry	GLC
*p*_sat_/kPa	0.036	0.509	0.509
$\Delta_{l}^{g}H_{m}$ exp.	**56.38**	**47.16**	47.45
	**56.11**	**47.29**	46.31
	**56.22**	**46.21**	46.80
	**56.30**		46.56
			46.60
$\Delta_{l}^{g}H_{m}$ avg.	56.25 ± 0.12	46.89 ± 0.59	46.65 ± 0.45
Mean rel. dev.	±0.20%	±1.26%	±0.97%

^a^ Data points printed in bold were used in the SimCor method (Section 2.6).

**Table 6:** Standard molar thermodynamic functions (in J·K^−1^·mol^−1^) of amides in the ideal gaseous state at *p* = 10^5^ Pa ^a^.

	*N*-Methylformamide				*N*,*N*-Dimethylformamide			
*T*/K	$C_{p,m}^{g0}$	$S_{m}^{g0}$	$\Delta_{0}^{T}H_{m}^{g0}/T$	$-\Delta_{0}^{T}G_{m}^{g0}/T$	$C_{p,m}^{g0}$	$S_{m}^{g0}$	$\Delta_{0}^{T}H_{m}^{g0}/T$	$-\Delta_{0}^{T}G_{m}^{g0}/T$
100	42.5	234.6	38.6	196.0	53.6	242.7	40.4	202.3
150	49.2	253.0	40.9	212.2	65.4	266.8	46.9	219.9
200	56.3	268.1	43.5	224.6	74.5	286.9	52.7	234.2
210	57.8	270.9	44.1	226.8	76.2	290.5	53.8	236.8
220	59.3	273.6	44.7	229.0	77.9	294.1	54.8	239.3
230	60.8	276.3	45.2	231.1	79.6	297.6	55.8	241.8
240	62.4	278.9	45.8	233.1	81.3	301.0	56.9	244.2
250	63.9	281.5	46.4	235.1	83.1	304.4	57.9	246.5
250	63.9	281.5	46.4	235.1	83.1	304.4	57.9	246.5
260	65.5	284.0	47.0	237.0	84.8	307.7	58.9	248.8
270	67.1	286.5	47.6	238.9	86.7	310.9	59.9	251.1
273.15	67.6	287.2	47.8	239.4	87.2	311.9	60.2	251.7
280	68.7	289.0	48.2	240.7	88.5	314.1	60.9	253.2
290	70.3	291.4	48.9	242.5	90.4	317.3	61.9	255.4
298.15	71.6	293.3	49.4	243.9	91.9	319.7	62.6	257.1
300	71.9	293.8	49.5	244.3	92.3	320.4	62.8	257.5
310	73.5	296.2	50.2	246.0	94.2	323.4	63.8	259.6
320	75.1	298.5	50.8	247.7	96.2	326.4	64.8	261.6
330	76.7	300.8	51.5	249.4	98.2	329.4	65.8	263.6
340	78.4	303.2	52.1	251.0	100.2	332.4	66.8	265.6
350	80.0	305.4	52.8	252.6	102.2	335.3	67.8	267.6
360	81.6	307.7	53.5	254.2	104.2	338.2	68.7	269.5
370	83.2	310.0	54.2	255.8	106.2	341.1	69.7	271.4
380	84.7	312.2	54.9	257.3	108.3	344.0	70.7	273.3
390	86.3	314.4	55.6	258.8	110.3	346.8	71.7	275.1
400	87.9	316.6	56.3	260.3	112.3	349.6	72.7	276.9
500	102.4	337.8	63.5	274.3	131.9	376.8	82.6	294.2
600	115.0	357.5	70.6	286.9	149.6	402.5	92.3	310.1
700	125.8	376.0	77.4	298.6	165.0	426.7	101.6	325.1
800	135.0	393.4	83.8	309.6	178.3	449.6	110.4	339.2
900	142.9	409.8	89.8	320.0	189.8	471.3	118.6	352.7
1000	149.8	425.2	95.3	329.9	199.7	491.8	126.2	365.6

^a^ Values were calculated with B3LYP-D3/6-311+G(2df,p) level of theory, as described in text. Values are reported with one digit more than is justified by the experimental uncertainty to avoid round-off errors in calculations based on these results.

**Table 7:** Parameters of the Cox equation, Equation (1).

Compound	*A*_0_	*A*_1_·10^3^	*A*_2_·10^6^	*T*^ref^/K	*p*^ref^/Pa ^a^	(*T*_min_ – *T*_max_)/K	*σ_p_*/Pa ^b^
*N*-Methylformamide	2.855705 ±0.001252	−1.067282 ±0.010432	0.795317 ±0.025846	472.346 ±0.090	100,000	248–473	152
*N*,*N*-Dimethylformamide	2.852686 ±0.000935	−1.597511 ±0.008325	1.572920 ±0.020373	425.101 ±0.026	100,000	215–427	441

^a^ *p*^ref^ was not considered an adjustable parameter, but was set to a constant value, ^b^ *σ_p_* is the standard deviation of the fit defined as $\sigma_{p}={\left[\sum_{i=1}^{n}{\left(\Delta p\right)}_{i}^{2}/\left(n-m\right)\right]}^{1/2}$, where Δ*p* is the difference between the experimental and the smoothed values, *n* is the number of experimental points used in the fit, and *m* is the number of adjustable parameters of the Cox equation.

**Table 8:** Sample description.

Compound	CAS Number	Supplier	Purification Method	Mole Fraction Purity	Mass Fraction Water Content
*N*-Methylformamide	123-39-7	Aldrich	Distillation, molecular sieves	0.988 ^a^; 0.9990 ^b^	30·10^−6 d^
*N*,*N*-Dimethylformamide	68-12-2	Aldrich	Vapor pressure measurements	0.9999 ^a^; 1.0000 ^b,c^	30·10^−6 e^

^a^ From certificate of analysis supplied by the manufacturer determined by gas-liquid chromatography (GLC). ^b^ Purity determined by GLC using the chromatograph Hewlett-Packard 6890A equipped with a column HP-1, length 25 m, film thickness 0.52 µm, diameter 0.32 mm, and an FID detector. Average of two determinations. ^c^ No detectable peaks found. ^d^ Fraction of water determined by Karl-Fischer analysis by Metrohm 831. Average of four determinations. ^e^ From certificate of analysis supplied by the manufacturer; determined by coulometry.

## Data Availability

The data presented in this study are available in the Supplementary Materials.

## Supplementary Materials

The following supporting information can be downloaded at: https://www.mdpi.com/article/10.3390/molecules29051110/s1. Thermogravimetric analysis for *N*-methylformamide (includes Figure S1), Analysis of literature enthalpies of vaporization (includes Table S1), Liquid heat capacities of *N*-methylformamide (comparison of heating and cooling regime, includes Figure S2), Thermodynamic relations used in simultaneous treatment of vapor pressures and related thermal data (SimCor method) (includes Table S2), Recommended Vaporization Enthalpies (includes Table S3).
