# Supplementary material for: Thermodynamic Study of N-Methylformamide and N,N-Dimethyl-Formamide
Source: Molecules. 2024 Mar 1;29(5):1110. doi: 10.3390/molecules29051110 (PMC10934579; doi:10.3390/molecules29051110)
Supplement: Supplementary file 1 [file molecules-29-01110-s001.zip › molecules-2891403-supplementary.pdf]

molecules

Supplementary Materials

Thermodynamic study of *N*-methylformamide and *N*,*N*-dimethyl-formamide

Květoslav Růžička [1,*], Vojtěch Štejfa [1], Ctirad Červinka [1], Michal Fulem [1] and Jiří Šturala [2]

[1] Department of Physical Chemistry, University of Chemistry and Technology, Prague, Technická 5, CZ-166 28 Prague, Czech Republic; stejfav@vscht.cz (V.Š.); cervinkc@vscht.cz (C.Č.); fulemm@vscht.cz (M.F.)
[2] Department of Inorganic Chemistry, University of Chemistry and Technology, Prague, Technická 5, CZ-166 28 Prague, Czech Republic; sturalaj@vscht.cz
* Correspondence: ruzickak@vscht.cz

Supplementary materials contain the following:

S1) Thermogravimetric analysis for *N*-methylformamide (contains Figure S1).
S2) Analysis of literature enthalpies of vaporization (contains Table S1).
S3) Liquid heat capacities of *N*-methylformamide (comparison of heating and cooling regime, contains Figure S2).
S4) Thermodynamic relations used in simultaneous treatment of vapor pressures and related thermal data (SimCor method) (contains Table S2).
S5) Recommended Vaporization Enthalpies (contains Table S3).

S1. Thermogravimetric analysis for *N*-methylformamide

Thermogravimetry used in this work for *N*-methylformamide is described in Section 3.2. and results for the heating rate 2 K min^{-1} and related discussion are presented in Section 2.1. Figure S1 shows results for the heating rate 5 K min^{-1}.

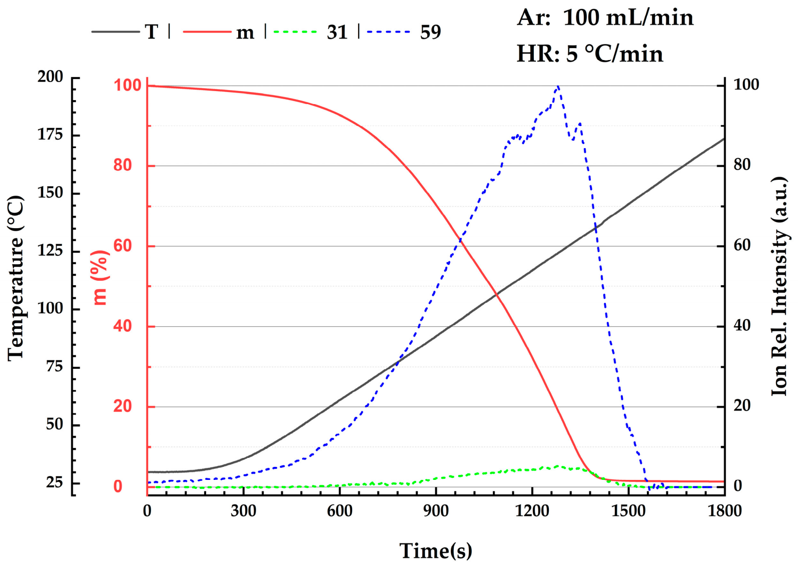

Figure S1. TG-MS spectrum of *N*-methylformamide at heating rate 5 K min^{-1}. Black solid line is temperature, red solid line is mass, dashed green line is NMF ion with m/z 31, and dashed blue line is NMF ion with m/z 59.

S2. Analysis of literature enthalpies of vaporization

The vaporization enthalpies are discussed in Section 2.3 in the main text; some additional details are provided here.

Barone et al. [49] introduced a modified commercial calorimeter for the determination of vaporization enthalpies, which was later employed by the same team to determine vaporization enthalpies of both *N*-methylformamide and *N,N*-dimethylformamide [16]. We found it useful to scrutinize the results obtained from testing the calorimeter [49] in order to estimate the uncertainty of calorimetric results for amides [16]. Six compounds were selected for testing (see Table S1) by Barone et al. [49]. Measurements were conducted as follows: after equilibrating for (1 to 3) hours subsequent to the insertion of the effusion vessel with the sample into the calorimeter, the sample was evaporated for (2 to 4) hours using a vacuum pump. The vessel was weighed before and after measurement to determine the mass of the evaporated sample.

Recommended values for four compounds were established by Majer and Svoboda [50] (within the framework of an IUPAC-supervised project) shortly after the presentation of the calorimeter [49]. For the remaining compounds (water and heavy water), the data can be compared with state-of-the-art equations of state published later by Wagner and Pruß [52] and Herrig et al. [51]. Absolute and relative deviations of the data by Barone et al. [49] from recommended data are given in the last two rows of Table S1. Given the fact that evaporation was unrestricted (i.e., equilibrium was not ensured), the results are surprisingly good. Note that the difference between equilibrium and non-equilibrium evaporation can amount to a product $R{\cdot}T$, i.e., 2.5 kJ·mol^{-1} [49]; deviations from reference data are significantly smaller even in the case of rather volatile benzene.

Table S1 Comparison of calorimetric results (in kJ·mol^{-1}) for reference compounds reported by Barone et al. [49] with recommended values.

Compound	Benzene	2-propanol	water	1-propanol	D_2O	1-butanol
p_{sat} /kPa [a]	12.69	5.78	3.17	2.81	2.74	0.90
ΔH_{vap} exp.	34.27	45.7	43.57	47.4	45.39	52.04
	34.64[b]	45.53	43.75	48.5	45.73	52.74
	34.02	45.32	43.82	47.46		51.31
			43.90	46.98		51.25
						53.94
ΔH_{vap} avg [49]	34.31±0.31	45.52±0.19	43.76±0.14	47.59±0.65	45.56±0.24	52.26±1.12
ΔH_{vap} rec. [50]	33.83 ±0.08	45.39 ±0.11	44.06 ±0.08[c]	47.45 ±0.12	45.50 ±0.11[d]	52.35 ±0.13
Abs. dev.	0.48	0.13	-0.30	0.13	0.06	-0.09
Rel. dev /%	1.42	0.28	-0.68	0.28	0.14	0.18

[a] Saturated vapor pressure. [b] This value is misprinted in Barone et al. [49] as 36.64.

[c] Recommended vaporization enthalpy calculated via Clausius Clapyroen equation from vapor pressure equation published by Wagner and Pruß [52]. Calorimetric values listed in Barone et al. [49] span from 43.98 kJ·mol^{-1} to 44.06 kJ·mol^{-1} and vaporization enthalpy calculated from EOS by Wagner and Pruß [52] is 43.99 kJ·mol^{-1}.

[d] Recommended vaporization enthalpy calculated via Clausius Clapyroen equation from vapor pressure equation published by Herrig et al. [51].

Vapor pressure for *N*-methylformamide at 298 K is approximately 0.035 kPa, lower than for the testing compound. While effusion cells with orifices ranging from 0.1 mm to 0.2 mm were used for testing compounds and for *N,N*-dimethylformamide (p_{sat}(298 K)=0.5 kPa), larger ones

(0.3 mm and 0.6 mm) were employed for *N*-methylformamide. There was an open question whether the results for compounds with low vapor pressure would also be reliable.

S3. Liquid heat capacities of *N*-methylformamide (comparison of heating and cooling regime)

Section 2.4 in the main article deals with liquid heat capacities. Unusual results were obtained in this work while heating *N*-methylformamide previously supercooled to 245 K instead of the standard 235 K. In this case, *N*-methylformamide did not solidify, and subsequent continued heating of the supercooled liquid resulted in a non-monotonous temperature dependence of heat capacity. The results of measurement in the cooling and heating mode are compared in Figure S2.

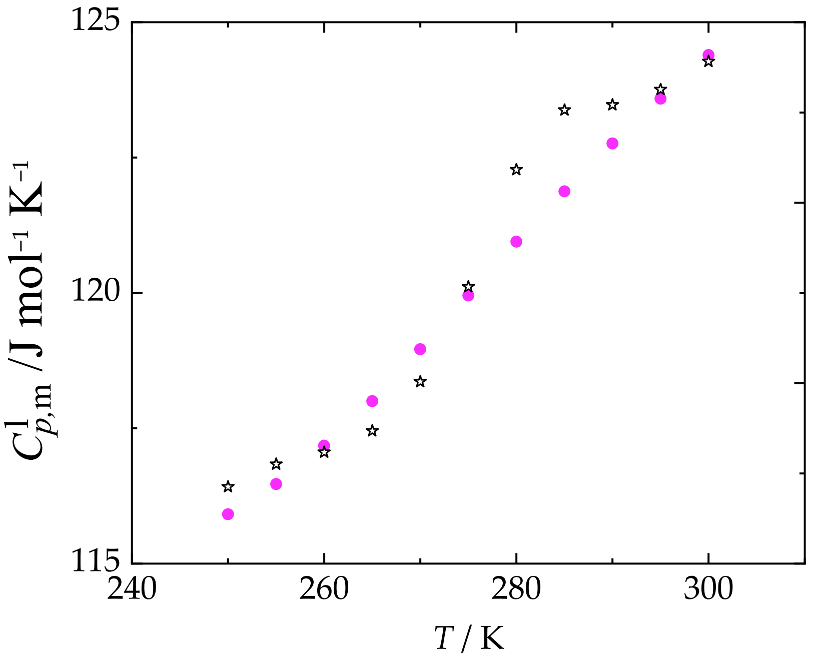

Figure S2. Liquid heat capacity of *N*-nethylformamid determined using calorimeter of Tian–Calvet type (SETARAM Microcalvet). Magenta ●, cooling mode; black ☆, heating mode.

This interesting (and reproducible) phenomenon would merit further investigation, for example, the results for supercooling to temperatures other than 235 K and 245 K might shed more light on the observed phenomenon. Such investigation is, however, out of the scope of this paper.

S4. Thermodynamic relations used in simultaneous treatment of vapor pressures and related thermal data (SimCor method)

The Simcor method was described previously in detail [76,98], and only the thermodynamic relations necessary to its understanding are repeated here for the reader's convenience.

Let us define auxiliary quantities $\Delta H'$ and $\Delta C'$:

$$\Delta H' \equiv \frac{\Delta_l^g H_m}{\Delta_l^g z} \tag{S1}$$

$$\Delta C' \equiv \left(\frac{d\Delta H'}{dT}\right)_{sat} = \frac{\Delta_l^g C_{p,m} - 2\Delta H'\left(\frac{\partial \Delta_l^g z}{\partial T}\right)_p - \frac{p}{RT}\Delta H'^2\left(\frac{\partial \Delta_l^g z}{\partial p}\right)_T}{\Delta_l^g z} \tag{S2}$$

where $\Delta_l^g z$ stands for the difference between the compressibility factors of the coexisting liquid and gas phases, $\Delta_l^g H_m$ is the vaporization enthalpy, and $\Delta_l^g C_{p,m} = C_{p,m}^g - C_{p,m}^l$ is the difference between isobaric heat capacity of gas and that of liquid phase at the saturation curve, i.e., at the vapor pressure p (the subscript 'sat' denotes a derivative along the saturation line; R is the molar gas constant).

The SimCor then starts from the Clapeyron equation in the form

$$\Delta H' = RT^2\left(\frac{d\ln p}{dT}\right)_{sat} \tag{S3}$$

which relates the vapor pressure p to $\Delta_l^g H_m$ and the pVT behavior of the coexisting phases. The differentiation yields an equation relating vapor pressures to heat capacities.

$$\Delta C' = R\left[\frac{d}{dT}T^2\left(\frac{d\ln p}{dT}\right)\right]_{sat} = 2RT\left(\frac{d\ln p}{dT}\right)_{sat} + RT^2\left(\frac{d^2\ln p}{dT^2}\right)_{sat} \tag{S4}$$

It is apparent that quantities $\Delta H'$ and $\Delta C'$ can be calculated exclusively from the vapor pressure equation (eqs S3 and S4) or from thermal properties ($\Delta_l^g H_m$ and $\Delta_l^g C_{p,m}^0$) and appropriate pVT corrections (eqs S1 and S2).

While calorimetry is a source of $\Delta_l^g H_m$ and $C_{p,m}^l$, the heat capacity of real gas is obtained using

$$C_{p,m}^g = C_{p,m}^{g0} - T\int_0^{p_{sat}}\left(\frac{\partial^2 V_m^g}{\partial T^2}\right)dp \tag{S5}$$

where $C_{p,m}^{g0}$ is isobaric heat capacity of ideal gas (see sections 2.5 and 3.5), and V_m^g is molar volume of gas phase.

Generally, the pVT term in equation S1 (i.e. term $\Delta_l^g z$) represents correction around (3 to 7) percent to $\Delta_l^g H_m$ at the normal boiling point temperature T_{nbp}, while pVT terms in equation S2 can amount up to 40 percent of $\Delta_l^g C_{p,m}$ value at T_{nbp}. Since data for exact evaluation of pVT corrections are generally not available, this correction is expressed by means of second virial coefficients (molar volumes of liquid phase can be neglected). As experimental second virial coefficients are typically

not available for temperatures well below the normal boiling temperature T_{nbp}, estimation methods must be used. This means that the uncertainty of *pVT* corrections is high and limits inclusion of thermal properties in the SimCor. Thus, to avoid the distortion of SimCor by errors in *pVT* description, $\Delta_l^g H_m$ can be included in the SimCor at saturated pressures smaller than approximately 10 kPa and heat capacity difference $\Delta_l^g C_{p,m}$ at saturated pressures less than approximately 1 kPa.

When the volume of gaseous phase is expressed as $V_m^g = \frac{RT}{p} + B$, eq S1 can be written as

$$\Delta H' = \frac{\Delta_l^g H_m}{1 + \frac{p_{sat}}{RT}\left(B - V_m^l\right)} \tag{S6}$$

and eq S2 can be converted (after neglecting the pressure dependence of V_m^l) to the form [96]

$$\Delta C' = \Delta_l^g C_{p,m}^0 - T\frac{d^2 B}{dT^2} p_{sat} - 2T\frac{d\left(B - V_m^l\right)}{dT}\left(\frac{dp}{dT}\right)_{sat} - T\left(B - V_m^l\right)\left(\frac{d^2 p}{dT^2}\right)_{sat} \tag{S7}$$

The molar volume of saturated liquid phase V_m^l and its temperature derivative play a negligible role at temperatures well below the normal boiling temperature, where equations S6 and S7 are applied and were therefore neglected.

Several estimation methods for the second virial coefficient can be found in the literature. We prefer the method suggested by Tsonopoulos [77], as it also provides (empirical) corrections for polar compounds. Input parameters for this estimation method are given in Table S2.

Table S2. Parameters Used for Evaluation of the Second Virial Coefficients by Tsonopoulos' Method [77].

compound	T_c/ K[a]	p_c/MPa[a]	ω[b]	μ / D
N-methylformamide	715.6	6.08	0.486	3.86 [79]
N,N-dimethylformamide	650.6	4.72	0.362	3.86 [80]

[a] critical temperature and pressure published by Wilson et al. [78].
[b] acentric factor calculated by the SimCor.

Note that in the limits suggested for applying *pVT* correction (p_{sat}<10 kPa in eq S6 and p_{sat}<1 kPa in eq S7), even a large change in input parameters would not influence resulting vapor pressures and enthalpies of vaporization.

In the present work, $\Delta H'$, evaluated using eq S6, was included in the SimCor at 298 K, where the vapor pressures of *N*-methylformamide and *N,N*-dimethylformamide are 0.035 kPa and 0.5 kPa, respectively.

The temperature range from the triple point to 300 K for both *N*-methylformamide and *N,N*-dimethylformamide, in which $\Delta C'$ (evaluated using eq S7) was included in the SimCor, corresponds to vapor pressures below the limit 1 kPa, in which *pVT* correction can be applied without affecting the final results.

S5. Recommended Vaporization Enthalpies

The enthalpy of vaporization can be calculated using the Cox equation, eq 1, with parameters given in Table 7 and *pVT* behavior using eq S6. This requires the calculation of the second virial coefficient *B* by using the Tsonopoulos method. To avoid the necessity of performing such calculations, recommended values of $\Delta_{cd}^{g}H_{m}$ are tabulated in Table S3 at discrete temperatures together with their estimated uncertainties.

Table S3. Recommended enthalpies of vaporization of *N*-methylformamide and *N,N*-dimethylformamide.

T / K	$\Delta_{l}^{g}H_{m}$ / kJ·mol^{-1}	
	N-methylformamide	*N,N*-dimethylformamide
240		49.75±0.23
250	59.29±0.29	49.13±0.24
260	58.77±0.30	48.53±0.25
270	58.24±0.31	47.93±0.26
280	57.72±0.33	47.34±0.28
290	57.20±0.34	46.76±0.29
298.15	56.79±0.36	46.37±0.31
300	56.68±0.36	46.18±0.31
310	56.16±0.38	45.61±0.32
320	55.64±0.40	45.05±0.35
330	55.13±0.42	44.50±0.37
340	54.61±0.44	43.95±0.39
350	54.09±0.47	43.40±0.41
360	53.58±0.50	42.86±0.44
370	53.05±0.53	42.32±0.47
380	52.53±0.56	41.79±0.49
390	52.00±0.60	41.26±0.52
400	51.46±0.63	40.73±0.55
410	50.92±0.67	40.20±0.58
420	50.37±0.71	39.67±0.60
430	49.81±0.75	
440	49.24±0.79	
450	48.66±0.83	
460	48.07±0.87	
470	47.47±0.91	

[a] The stated uncertainties reflect the uncertainties of the input data. The SimCor method has been used several times while varying the input data by their uncertainties. The stated expanded uncertainties (0.95 level of confidence) are the resulting variations of the fitted values for vaporization enthalpies.
